# Metastatic deaths in retinoblastoma patients treated with intraarterial chemotherapy (ophthalmic artery chemosurgery) worldwide

**DOI:** 10.1186/s40942-017-0093-8

**Published:** 2017-10-23

**Authors:** David H. Abramson, Carol L. Shields, Pascal Jabbour, Luiz Fernando Teixeira, José Roberto Falco Fonseca, Marcio Chaves Pedro Marques, Francis L. Munier, Francesco Puccinelli, Theodora Hadjistilianou, Sandra Bracco, Guillermo Chantada, Alejandro Ceciliano, Y. Pierre Gobin

**Affiliations:** 1 Department of Surgery, Memorial Sloan Kettering Cancer Center, New York, NY 10065 USA; 2 Department of Ophthalmology, Weill-Cornell Medical School New York, New York, NY 10065 USA; 3 Ocular Oncology Service, Wills Eye Hospital, Thomas Jefferson University, Philadelphia, PA 19107 USA; 4 Department of Neurovascular and Endovascular Surgery, Department of Neurological Surgery, Thomas Jefferson University, Philadelphia, PA 19107 USA; 5 Department of Ophthalmology, Federal University of São Paulo (UNIFESP), São Paulo, 04021-001 Brazil; 6 Diagnostic Imaging Department, Pediatric Oncology Institute, Federal University of São Paulo (UNIFESP), São Paulo, 04021-001 Brazil; 7 Pediatric Oncology Department, Santa Marcelina Hospital, São Paulo, 03953-120 Brazil; 8 Unit of Pediatric Ocular Oncology, Jules-Gonin Eye Hospital, University of Lausanne, 1015 Lausanne, Switzerland; 9 Interventional Neuroradiology Unit, Department of Radiology, Centre Hospitalier Iniversitaire Vaudois, 1011 Lausanne, Switzerland; 10 Department of Surgery, University of Siena, 53100 Siena, Italy; 11 Neurological and Sensorineural Department, Azienda Universitaria Ospedaliera Sense, 53100 Siena, Italy; 12 Hematology Oncology Service, Hospital JP Garrahan, 1800 Buenos Aires, Argentina; 13 Interventional Radiology, Hospital Universitario Austral, 1500 Buenos Aires, Argentina; 14 Department of Radiology and Neurosurgery, New York Presbyterian Hospital, New York, NY 10065 USA

**Keywords:** Intra-arterial (intrarterial) chemotherapy (IAC), Metastatic death, Ophthalmic artery chemosurgery (OAC), Retinoblastoma (Rb), Melphalan

## Abstract

**Background:**

Ophthalmic artery chemosurgery [OAC, intra-arterial chemotherapy (IAC)] was introduced in 2006 as treatment modality for intraocular retinoblastoma. The purpose of this commentary is to retrospectively review the incidence of metastatic deaths in retinoblastoma patients treated with OAC worldwide over a 10 year period. Retrospective data regarding metastatic deaths was collected from six international retinoblastoma centers (New York City USA, Philadelphia USA, Sao Paulo Brazil, Siena Italy, Lausanne Switzerland and Buenos Aires Argentina). All retinoblastoma patients from these centers (naive and recurrent, unilateral and bilateral) treated with OAC/IAC since 2006 have been included in this study. Data regarding number of patients, number of OAC/IAC infusions, number unilateral and bilateral, number treated for naive disease or salvage and number of metastatic deaths have been assessed. Over a 10-year period of time 1139 patients received OAC/IAC for 4396 infusions. At last follow-up there were only three metastatic deaths (all treated in Buenos Aires).

**Conclusion:**

The current survey assessed the recorded risk of metastatic deaths in six retinoblastoma centers worldwide in children with retinoblastoma (unilateral or bilateral) treated with OAC/IAC as primary or secondary therapy. Overall, the observed risk for metastatic deaths from retinoblastoma was <1% in OAC/IAC treated children.

## Background

For more than 100 years, ocular oncologists have been perfecting and attempting to find better alternatives to enucleation for management of intraocular retinoblastoma. Small tumors without vitreous or subretinal seeding have been effectively treated with laser photocoagulation, thermotherapy, cryotherapy or brachytherapy without compromising patient survival from metastatic disease [1]. As far back as 1903 and continuing for almost 90 years, external beam radiotherapy was the only way to salvage eyes with advanced intraocular disease [2]. While there were no randomized trials comparing radiation to enucleation, it was universally recognized that conservative (non-enucleation) therapies lead to similar rates of metastatic death. In the 1990s, radiation was abandoned because of its impact on the development of second non-ocular cancers (which caused more deaths than primary retinoblastoma) [3] and was replaced with systemic chemotherapy [4]. In 1996, several papers described patients managed with systemic chemotherapy (with focal consolidation treatments) and they did not appear to have an increase in metastatic deaths (again-no randomized trials were performed) but difficulty with tumor control for advanced disease with this technique lead to the development of intra-arterial chemotherapy [IAC, ophthalmic artery chemosurgery (OAC)] for advanced retinoblastoma. In some centers using IAC/OAC, the enucleation rate has now dropped to 5% [5], but some authors have questioned if successful salvage of these eyes occurs with an increase in metastatic deaths from retinoblastoma [6]. Our six centers from the USA, Europe and Latin America have had extensive experience over a 10-year period with OAC/IAC and collaborated in the investigation of this query to report on the risk for metastatic deaths from retinoblastoma in children treated with IAC/OAC.

## Survey

A retrospective survey of all patients treated with OAC/IAC between May 2006 and November 1, 2016 from six Rb centers was performed. Metastatic death from retinoblastoma was reported in addition to tumor laterality, number of cases treated with OAC/IAC, number of infusions delivered in each center and whether the patients received OAC/IAC as primary treatment (naïve) or secondary (after prior systemic chemotherapy and or radiotherapy). Each center used similar technique with combinations of Melphalan, Topotecan and Carboplatin, but there were minor variations in medication combinations and number of cycles as this was a retrospective analysis.

Between May 1, 2006 and November 1, 2016, our six centers treated 1177 eyes of 1139 patients with 4396 separate OAC/IAC infusions. There were 781 unilateral and 358 bilateral cases. There were 464 (39%) eyes that were naïve and 713 (61%) treated after prior systemic chemotherapy and/or radiation. Of these 1139 patients, there were 3 (<1%) who died from metastatic retinoblastoma (Table 1).

**Table 1:** Summary data from 6 centers of all patients treated with OAC between May 2006 and November 1, 2016

Patients	Infusions	Uni/bilateral	First line (#eyes)	Salvage (# eyes)	Metastatic deaths
1139	4396	781/358	464	713	3

## Discussion

Ophthalmic artery chemosurgery (OAC)/intra-arterial chemotherapy (IAC) has become an important tool for intraocular retinoblastoma management. This technique was first performed by Reese et al. more than 60 years ago after delivering a nitrogen mustard derivative by direct puncture of the carotid artery. Despite a “dramatic” response, this strategy was abandoned because of complications (including one death from carotid bleeding) and one death from neutropenia. Reese never reported on metastatic deaths in the 42 patients with retinoblastoma managed with carotid artery infusion.

Beginning more than 40 years ago, the Japanese perfected a technique they called “selective ophthalmic artery infusion (SOAI)” of chemotherapy using Melphalan, a powerful alkylating agent that they had in vitro determined was the most potent drug available for retinoblastoma. They reported on tumor control in a cohort of 343 patients treated with several methods and found metastatic deaths in 8, 5 with metastases and 3 with direct or indirect extension into the central nervous system and death [7]. Unfortunately, it is impossible to know the exact treatment each child received because that report combined hundreds of retinoblastoma cases treated with external beam radiation, multiagent systemic chemotherapy, intravitreal chemotherapy (Melphalan) and hyperthermia in addition to SOAI, with approximately 50% of these patients having all of the above treatments. Despite this, the authors compared established death rates from retinoblastoma in Japan and concluded that there was no increase in deaths attributable to SOAI. The Japanese study cannot be directly compared to the current survey since follow-up is much more long term.

The modern intra-arterial approach was introduced by two of us (DHA, YPG) in New York in May, 2006 [8]. Since that time it has been taught and adopted by nearly all the major retinoblastoma sites worldwide [9–11], and in a recent published worldwide survey chosen as the first line approach for unilateral advanced retinoblastoma [12]. Several major retinoblastoma centers employ OAC/IAC for both naive and failed advanced eyes and in select cases where macula tumors threaten vision for both unilateral and bilateral disease. While differences in choosing indications for OAC/IAC exist, the technique, drugs, doses, frequency and catheters are generally the same in all centers [9]. With time and experience, we have achieved an eye salvage rate that improves with experience and now only 5–6% of all eyes with retinoblastoma are primarily enucleated as a result of using first line OAC/IAC and of those eyes treated 95% are retained [5, 13]. The frequency of metastatic deaths in OAC treated children has been reported by many centers worldwide and a year ago our centers in New York, Philadelphia, Argentina and Switzerland reported on 634 cases with only one metastatic death [9].

A recent publication (from authors who had never done the procedure) has raised the question about metastatic deaths [6]. These authors also questioned if leaving an eye in with higher risk pathological features might preclude giving systemic chemotherapy and therefore compromise survival. Since OAC/IAC has become a transformative option for the management of retinoblastoma from the time of radiation, we decided to update our collective experience and include two additional experienced centers-from Italy and Brazil to expand our knowledge base. There were no metastatic deaths from the two USA centers, two European centers or Brazil but 3 from the center in Argentina and they are instructive.

### Case 1

Bilateral retinoblastoma with a positive family history. The child was initially treated with systemic chemotherapy and progressed and received OAC. After progression intravitreal chemotherapy was given and then a second round of systemic chemotherapy which also failed. The family refused enucleation. Orbital invasion and metastasis with death ensued.

### Case 2

Bilateral retinoblastoma with a positive family history. One eye (International Classification E) refused enucleation and then refused systemic chemotherapy. The child was given OAC/IAC while social services worked to get consent for enucleation. Enucleation eventually performed and demonstrated scleral involvement and post laminar invasion. Family did not return for follow-up and then developed orbital disease that was treated with systemic chemotherapy but died after a CNS relapse.

### Case 3

Bilateral retinoblastoma with a positive family history. The child was initially treated with systemic chemotherapy and external beam irradiation. Because of progressive disease enucleation recommended but refused so OAC/IAC given. The disease progressed after treatment and enucleation was performed which showed disease at the cut margin of the optic nerve. Orbital and CNS invasion developed and later death.

Thus two of the deaths occurred in eyes previously treated with combinations of systemic chemotherapy and external beam irradiation and all deaths (2.6% of the patients treated in Argentina) occurred in families refusing enucleation. This is comparable to the previously reported experience in the same hospital in the pre-OAC era (2.3%) [14]. Refusal of enucleation occurs more frequently in less developed countries [15].

This report summarizes the 10-year experience from large retinoblastoma centers with experience with OAC/IAC. Although the centers do not collaborate on a prospective trial algorithm they are all using the same technique, doses, drugs and catheters introduced by us at Memorial Sloan Kettering Cancer Center and have all independently (and collectively) reported similar high ocular success rates. All metastatic deaths occurred in the Argentinian cohort and followed refusal of enucleation or poor follow up.

The finding that metastatic deaths from retinoblastoma are rare in patients treated with OAC/IAC is reassuring as this technique allows eyes that were previously enucleated worldwide to be salvaged.

## Conclusion

This retrospective survey reports on the incidence of metastatic deaths in retinoblastoma patients managed OAC/IAC. Over a 10-year period, children with retinoblastoma (unilateral or bilateral) treated with OAC/IAC as primary or secondary therapy showed <1% risk for metastatic deaths from retinoblastoma.

## Abbreviations

OAC: ophthalmic artery chemosurgery
IAC: intra-arterial chemotherapy
Rb: retinoblastoma
